# SARS-CoV-2 infection in the mouse olfactory system

**DOI:** 10.1038/s41421-021-00290-1

**Published:** 2021-07-06

**Authors:** Qing Ye, Jia Zhou, Qi He, Rui-Ting Li, Guan Yang, Yao Zhang, Shu-Jia Wu, Qi Chen, Jia-Hui Shi, Rong-Rong Zhang, Hui-Ming Zhu, Hong-Ying Qiu, Tao Zhang, Yong-Qiang Deng, Xiao-Feng Li, Jian-Feng Liu, Ping Xu, Xiao Yang, Cheng-Feng Qin

**Affiliations:** 1 State Key Laboratory of Pathogen and Biosecurity, Beijing Institute of Microbiology and Epidemiology, Beijing, China; 2 State Key Laboratory of Proteomics, National Center for Protein Science (Beijing), Beijing Institute of Lifeomics, Beijing, China; 3 Department of Otorhinolaryngology, China-Japan Friendship Hospital, Beijing, China; 4 Research Unit of Discovery and Tracing of Natural Focus Diseases, Chinese Academy of Medical Sciences, Beijing, China

**Keywords:** Mechanisms of disease, Cell signalling

## Abstract

SARS-CoV-2 infection causes a wide spectrum of clinical manifestations in humans, and olfactory dysfunction is one of the most predictive and common symptoms in COVID-19 patients. However, the underlying mechanism by which SARS-CoV-2 infection leads to olfactory disorders remains elusive. Herein, we demonstrate that intranasal inoculation with SARS-CoV-2 induces robust viral replication in the olfactory epithelium (OE), not the olfactory bulb (OB), resulting in transient olfactory dysfunction in humanized ACE2 (hACE2) mice. The sustentacular cells and Bowman’s gland cells in the OE were identified as the major target cells of SARS-CoV-2 before invasion into olfactory sensory neurons (OSNs). Remarkably, SARS-CoV-2 infection triggers massive cell death and immune cell infiltration and directly impairs the uniformity of the OE structure. Combined transcriptomic and quantitative proteomic analyses revealed the induction of antiviral and inflammatory responses, as well as the downregulation of olfactory receptor (OR) genes in the OE from the infected animals. Overall, our mouse model recapitulates olfactory dysfunction in COVID-19 patients and provides critical clues for understanding the physiological basis for extrapulmonary manifestations of COVID-19.

## Introduction

The emergence of coronavirus disease 2019 (COVID-19), caused by the newly identified severe acute respiratory syndrome coronavirus 2 (SARS-CoV-2), has led to a global crisis. The clinical manifestations of SARS-CoV-2 infection predominantly involve the respiratory system, including cough, sore throat, pneumonia, and acute respiratory distress syndrome (ARDS)^1,2^. As the disease continues to spread widely, a significant portion of COVID-19 patients are developing anosmia, hyposmia, or other olfactory dysfunctions according to clinical reports^3–5^. Accumulated evidence has established the alteration of smell as one of the most predictive symptoms for COVID-19 screening^5,6^.

The perception of smell begins with odorant binding to the olfactory receptors (ORs) of olfactory sensory neurons (OSNs) along the upper surface of the olfactory epithelium (OE). Each OSN projects an axon into the glomerulus of the olfactory bulb (OB) and then synapses with second-order neurons to convey odour information to the olfactory cortex. Previously, upper respiratory tract infections were considered a common cause of olfactory disorders. Mouse models have been used to reproduce olfactory system infection and subsequent olfactory dysfunction^7,8^. For example, post viral olfactory disorders were observed in Sendai virus-infected mice by the buried food pellet test (BFPT), as well as the impairment of OE and OB tissues^9^. However, an animal model that can recapitulate the olfactory dysfunctions seen in COVID-19 patients remains lacking.

Human nasal respiratory epithelium (RE) cells exhibit high expression of angiotensin-converting enzyme 2 (ACE2)^10,11^, the functional receptor of SARS-CoV-2^12–14^. Single-cell RNA sequencing analyses have characterized the expression profile of ACE2 in the OE of mice and humans, mainly in non-neuroepithelial cells^11,15^, and in a recent study based on a hamster model, many SARS-CoV-2-infected cells were observed in the OE section^16,17^. In addition, vascular pericytes in the OB exhibited a high level of ACE2 expression in a mouse model^15^; these cells play a key role in the maintenance of the blood–brain barrier, as well as the regulation of blood pressure and the host immune response^18^. Interestingly, some respiratory viruses, such as influenza virus and respiratory syncytial virus, are able to invade the OB and other parts of the brain to establish infection^19,20^. Thus, how SARS-CoV-2 invades the olfactory system and contributes to the observed central nervous system (CNS) diseases remains to be determined. In the present study, we demonstrate that SARS-CoV-2 infection directly causes transient olfactory dysfunction in an established mouse model and characterize the major target cells and pathological effects that contribute to olfactory dysfunction.

## Results

### SARS-CoV-2 targets the OE and causes transient olfactory dysfunction in hACE2 mice

**Fig. 1:**
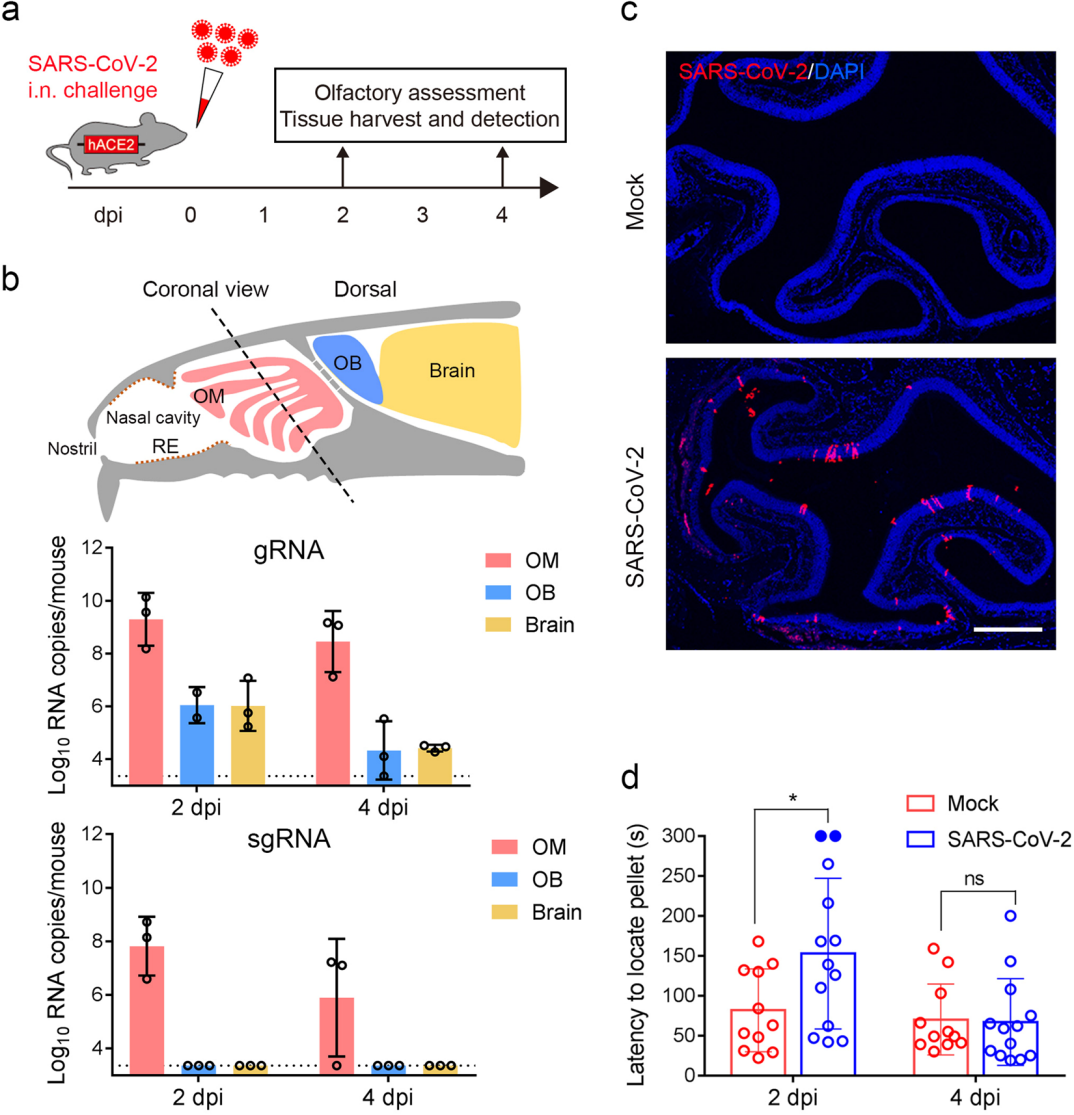
SARS-CoV-2 primarily infects the OE and causes olfactory dysfunction in hACE2 mice. **a** Schematic diagram of the experimental design. Briefly, groups of 6–8-week-old hACE2 mice were infected with 5.4 × 10^5^ PFU of SARS-CoV-2 intranasally. The olfactory function of infected mice was measured by the buried food pellet test at the indicated times post inoculation. Mice were sacrificed at 2 dpi and 4 dpi for viral detection and histopathological analysis. **b** Schematic view of the OM in the nasal cavity of mice in a sagittal plane. The dotted line indicates a coronal section (upper). Viral genomic RNA (gRNA, middle) and subgenomic RNA (sgRNA, lower) copies were determined by quantitative real-time reverse transcription PCR (qRT-PCR) and are shown as means ± SD from three independent replicates. **c** Immunostaining of the OM from SARS-CoV-2-infected mice for SARS-CoV-2 N protein (red) and DAPI (blue) at 2 dpi. Scale bar, 400 μm. **d** Buried food pellet test. The latency to locate the food pellets for mice infected with SARS-CoV-2 (*n* = 13) or DMEM (*n* = 11) was measured at 2 dpi and 4 dpi.

We previously established a humanized ACE2 (hACE2) mouse model susceptible to SARS-CoV-2 infection^21^. Herein, to determine the impact of SARS-CoV-2 infection on the olfactory system, groups of 6–8-week-old hACE2 mice were intranasally infected with 5.4 × 10^5^ plaque-forming units (PFU) of SARS-CoV-2. Mice inoculated with the same volume of culture medium were used as mock infection controls. At 2 and 4 days post infection (dpi), tissues from the respiratory tract and olfactory system were collected from the necropsied mice and subjected to virological and immunological assays (Fig. 1a). As expected, high levels of SARS-CoV-2 RNA was detected in the nasal respiratory RE, trachea, and lung at 2 and 4 dpi, and the peak viral RNA level (2.36 × 10^11^ RNA copies/mouse) was detected in the lung at 2 dpi (Supplementary Fig. S1a), while robust viral nucleocapsid (N) protein expression was detected in the lung from SARS-CoV-2-infected hACE2 mice at 2 dpi and 4 dpi but not in the control animals (Supplementary Fig. S1b). Strikingly, high levels of viral genomic RNA (gRNA) were also detected in the olfactory mucosa (OM) at 2 dpi (5.85 × 10^9^ RNA copies/mouse) and maintained until 4 dpi (8.93 × 10^8^ RNA copies/mouse) (Fig. 1b), while the viral RNA levels were much lower in the OB and other parts of the brain at 2 dpi and decreased to marginal levels at 4 dpi. Additionally, high levels of viral subgenomic RNA (sgRNA) (2.24 × 10^8^ RNA copies/mouse), indicating the presence of actively replicating virus, were detected in the OM at 2 dpi, and subsequently decreased at 4 dpi (9.89 × 10^6^ RNA copies/mouse); however, the sgRNA levels in the OB and brain were below the detection threshold (Fig. 1b). As expected, in situ hybridization (ISH) by RNAscope demonstrated that SARS-CoV-2 RNA was predominantly detected in OE (Supplementary Fig. S2a) but not in the OB (Supplementary Fig. S2b). Furthermore, an immunofluorescence staining assay detected high levels of SARS-CoV-2 N protein expression in the OE along the OM at 2 dpi (Fig. 1c), and only marginal expression of viral protein was observed at 8 dpi (Supplementary Fig. S3). However, we did not detect SARS-CoV-2 N protein-positive cells in the OB or other parts of the brain at 2 dpi and 4 dpi (Supplementary Fig. S2c).

To examine whether SARS-CoV-2 infection directly impairs the olfactory function of infected mice, a standard BFPT was conducted at 2 and 4 dpi. Remarkably, a significantly increased latency (152.8 s vs 81.8 s; *P* = 0.022) to locate food pellets was observed in SARS-CoV-2-infected mice compared with the control animals at 2 dpi (Fig. 1d). Of particular note, 2 out of the 13 infected mice developed severe symptoms of anosmia, as they failed to locate the food pellet within the observation period. Interestingly, recovery of infected mice from olfactory dysfunction was observed at 4 dpi, as the latency to locate food pellets was not different from that of the control animals (67.1 s vs 70.2 s; *P* = 0.992). Thus, these results demonstrate that SARS-CoV-2 primarily infects the OE and leads to transient olfactory dysfunction in mice.

### SARS-CoV-2 primarily targets non-neuroepithelial cells in the OE of hACE2 mice

The OM consists of the OE and the underlying lamina propria (LP). The OE is composed of olfactory stem/progenitor cells, including the horizontal basal cells (HBCs) and globose basal cells (GBCs) residing in the basal region, mature and immature OSNs, and a variety of non-neuroepithelial lineages, including the sustentacular cells, microvillar cells, and Bowman’s gland cells. The OSNs lining under the supporting cells project numerous dendritic cilia with ORs into the nasal cavity and intermingle with the microvilli of sustentacular cells and microvillar cells (Supplementary Fig. S4a). Due to the asymmetrical expression pattern of ACE2 on the cell membrane as well as the unique organization of the OE, it is not easy to determine which cell compartments express ACE2. To overcome this, we took advantage of the tdTomato cassette downstream of the hACE2 transgene with an internal ribosome entry site (IRES), which allows the detection of hACE2 expression by cytoplasmic fluorescence of tdTomato (Supplementary Fig. S4b). Abundant expression of hACE2 along the apical surface of the OE and within the underlying LP was detected with a human ACE2-specific monoclonal antibody, exhibiting a similar expression pattern as tdTomato (Supplementary Fig. S4c). A detailed characterization of hACE2/tdTomato-expressing cells in the OM revealed that non-neuroepithelial cells, including sustentacular cells (CK8 positive, Supplementary Fig. S4d, d1), the duct and acinus of Bowman’s gland cells (Sox9/CK8 positive, Supplementary Fig. S4d, d2, d4) in the OE and LP, respectively, and microvillar cells (CD73/CK8 positive, Supplementary Fig. S4e), are the primary cell types that exhibit human ACE2 expression (Supplementary Fig. S4d, f), whereas little hACE2/tdTomato expression was detected in the neuroepithelial lineages, including HBCs (CK5 positive), GBCs (Sox2 positive in the basal region), immature olfactory sensory neurons (iOSNs) (GAP43 positive), and mature olfactory sensory neurons (mOSNs) (OMP positive) (Supplementary Fig. S4d, d1–d4).

**Fig. 2:**
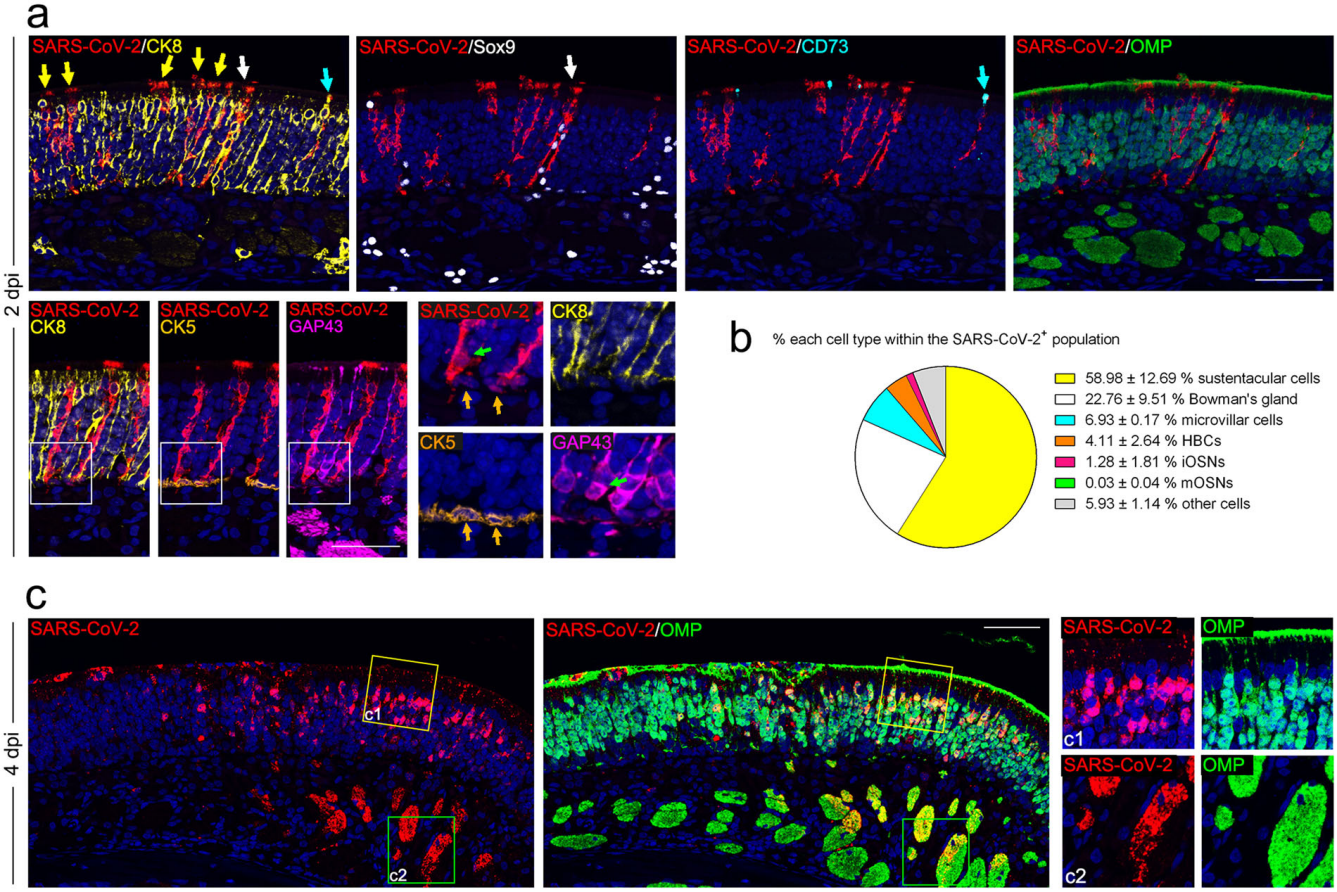
SARS-CoV-2 primarily targets non-neuroepithelial cells in the OE. **a** Representative multiplex immunofluorescent staining assay showing that SARS-CoV-2 (SARS-CoV-2 N protein positive) infects sustentacular cells (CK8 positive, yellow arrows), Bowman’s gland cells (Sox9/CK8 positive, white arrows), microvillar cells (CD73/CK8 positive, cyan arrows), HBCs (CK5 positive, gold arrows), and iOSNs (GAP43 positive, green arrows) at 2 dpi. Little SARS-CoV-2 N protein was detected within OMP-positive mOSNs. **b** Statistical analysis of the percentage of each cell compartment within SARS-CoV-2-positive cells. The data are presented as means ± SD (*n* = 3). **c** Multiplex immunofluorescent staining results showing an OM sample at 4 dpi with SARS-CoV-2 detected in the OMP-positive mOSNs and the underlying nerve bundles. The framed areas labeled **c**1 and **c**2 are shown adjacently at larger magnifications. Scale bar, 50 μm.

To further characterize the primary targets of SARS-CoV-2 in the OE, multiplex immunostaining assays were performed with antibodies against SARS-CoV-2 and specific cell markers. Remarkably, robust expression of SARS-CoV-2 viral N protein was detected in the non-neuroepithelial lineage lining the outer surface of the OE at 2 and 4 dpi (Fig. 2a, c). Sustentacular cells (58.98%) and Bowman’s gland cells (22.76%) were the major target cell types at 2 dpi, while some microvillar cells (6.93%) and HBCs (4.11%) were also infected (Fig. 2a, b). A small population of iOSNs (1.28%) was also infected by SARS-CoV-2, while no mOSNs were infected at 2 dpi (Fig. 2a, b). Interestingly, SARS-CoV-2-positive HBCs and iOSNs were found adjacent to the infected sustentacular cells (Fig. 2a). Additionally, a substantial amount of viral protein was detected within the cilia, cellular bodies, and underlying nerve bundles of mOSNs at 4 dpi (Fig. 2c, c1, c2). These results indicated that SARS-CoV-2 primarily targets the non-neuroepithelial cells lining the outer surface of the OE and subsequently invades the neuroepithelial lineage in hACE2 mice.

### SARS-CoV-2 infection triggers apoptosis and immune cell infiltration in the OE

**Fig. 3:**
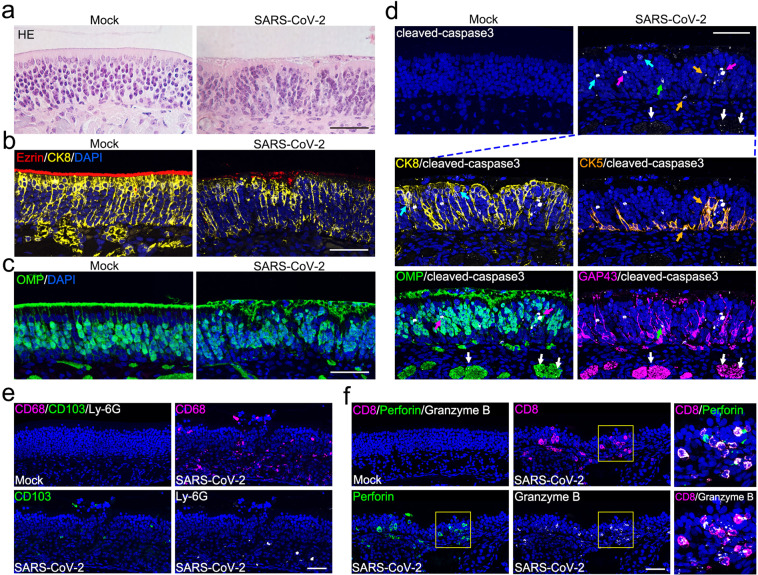
SARS-CoV-2 infection induces apoptosis and immune cell infiltration in the OE. **a** Representative hematoxylin and eosin (H&E) staining results showing histopathological changes of the OE. **b** Representative results of multiplex immunofluorescent detection of sustentacular cells (CK8 positive) and microvilli (Ezrin positive) of the OE. **c** Representative results of immunofluorescent detection of mOSNs (OMP positive) of the OE. **d** Apoptosis of olfactory epithelial cells (cleaved-caspase3 positive, white) after SARS-CoV-2 infection. The panels below show apoptosis of sustentacular cells (CK8 positive, yellow; indicated by cyan arrows), HBCs (CK5 positive, gold; indicated by gold arrows), mOSNs (OMP positive, green; indicated by magenta arrows), iOSN (GAP43 positive, magenta; indicated by green arrows), and olfactory nerve bundles (OMP/GAP43 positive; indicated by white arrows). **e** Representative multiplex immunofluorescent staining results showing infiltration of macrophages (CD68 positive, magenta), dendritic cells (CD103 positive, green), and neutrophils (Ly-6G positive, white) in the OE after infection. **f** Representative multiplex immunofluorescent staining results showing infiltration of CD8 cytotoxic T lymphocytes (magenta) with expression of perforin (green) and granzyme B (white) in the olfactory mucosa after infection. The framed areas are shown adjacently at larger magnifications. Scale bar, 50 μm.

We then characterized the histopathological changes in the OE in response to SARS-CoV-2 infection. Strikingly, SARS-CoV-2 infection directly impaired the structural uniformity of the OE, as characterized by clusters of remnants on the surface of the OE (Fig. 3a), as well as disorganized arrangement of supporting cells (Fig. 3b) and olfactory neurons (Fig. 3c). The integrity of the cilia layer of mOSNs and the microvilli of supporting cells were severely damaged (Fig. 3b, c). More importantly, compared with the mock-treated groups, profound cell apoptosis (cleaved-caspase3 positive) was observed in both the OE and LP sections of the OM from SARS-CoV-2-infected mice (Fig. 3d). Immunofluorescence co-staining indicated the occurrence of apoptosis in sustentacular cells, HBCs as well as the cellular bodies and underlying nerve bundles of iOSNs and mOSNs (Fig. 3d). Additionally, infiltration of immune cells, including macrophages (CD68 positive), dendritic cells (CD103 positive) and neutrophils (Ly-6G positive), was evident in the infected OE (Fig. 3e). The profound invasion of CD8 T lymphocytes with high expression of the cytotoxic enzymes perforin and granzyme B further deteriorated the cellularity of olfactory epithelial cells (Fig. 3f). This observed physiological damage that occurs upon SARS-CoV-2 infection probably contributes to the functional loss of olfaction.

### SARS-CoV-2 infection induces regeneration of the OE

Without infection, HBCs in the basal region of the OE remained quiescent, as indicated by the low expression level of the proliferation marker Ki67 within CK5-positive cells (Supplementary Fig. S5a, a1). SARS-CoV-2 infection significantly increased the number of CK5/Sox2/Ki67 triple-positive cells, strongly suggesting a transition from HBCs to actively cycling GBCs (Supplementary Fig. S5a, a2). Of particular note, prominent upward growth of HBCs from the basal layer into the upper section of the OE was observed in infected animals, which also co-expressed the markers of their lineage offspring, such as iOSNs (Supplementary Fig. S5b, b1), sustentacular cells (Supplementary Fig. S5b, b2), and microvillar cells (Supplementary Fig. S5b, b3). These results suggest that the impaired OE is regenerated through olfactory stem cell-based proliferation and differentiation into olfactory neurons and supporting lineages, thereby restoring the normal function of the OE.

### SARS-CoV-2 infection induces antiviral and inflammatory responses in the OE

**Fig. 4:**
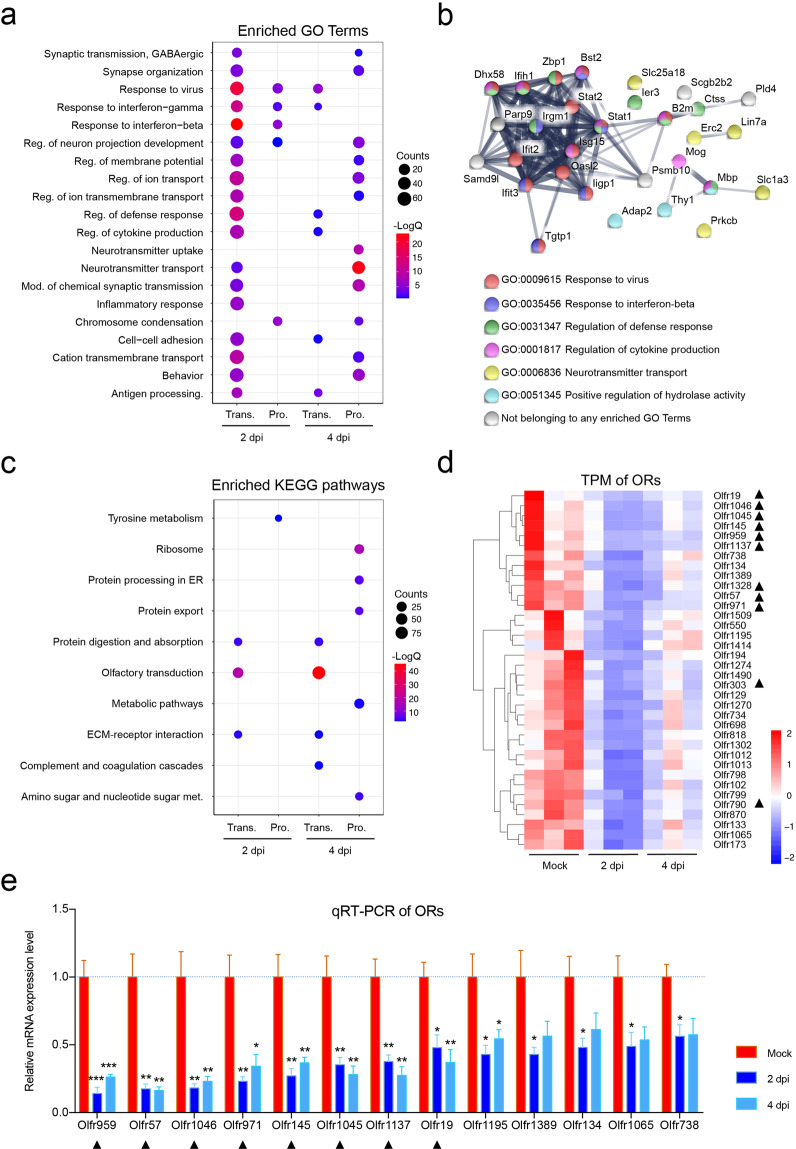
Host response to SARS-CoV-2 in the OE at the mRNA and protein levels. **a** Dot plot visualization of enriched GO terms of upregulated genes/proteins at 2/4 dpi in the OE. Gene enrichment analyses were performed using Metascape against the GO dataset for biological processes. “Reg.” stands for regulation, “mod.” for modulation, and “antigen processing.” for antigen processing and presentation of peptide antigen. **b** Interaction map of 30 proteins that were consistently upregulated at both the transcriptomic and proteomic levels over the course of SARS-CoV-2 infection in the OE. Network nodes represent proteins, and their colors indicate the different GO terms to which they belong. Edges represent protein–protein associations, and their thickness indicates the strength of data support. **c** Dot plot visualization of enriched KEGG pathways of downregulated genes/proteins at 2/4 dpi in the OE. Gene enrichment analyses were performed using STRING against the KEGG dataset. “Met.” for metabolism. The color of the dots represents the –LogQ value for each enriched KEGG pathway, and size represents the gene/protein counts enriched in each term. **d** Heatmap indicating the expression patterns of 36 olfactory receptor genes that were significantly downregulated at 2 dpi. The colored bar represents the *Z*-score of the TPM. A total of 11 genes that were also downregulated at 4 dpi are marked with black triangles. **e** RNA expression of 13 representative ORs by qRT-PCR. Columns with *, **, *** indicate ORs significantly downregulated at *P* < 0.05, *P* < 0.01, or *P* < 0.001, respectively, relative to their mock groups (one-way ANOVA followed by post hoc analysis with Dunnett’s multiple comparisons test, *n* = 3). Black triangle-marked ORs were downregulated at both 2 and 4 dpi based on transcriptome data.

To decipher the mechanism underlying the observed olfactory dysfunction in SARS-CoV-2-infected mice at the molecular level, combined transcriptomic and quantitative proteomic analyses of the OE and OB samples from SARS-CoV-2-infected mice were performed, and the results were compared with those of the control animals. In the OE samples, a total of 929 genes and 507 proteins were regulated upon SARS-CoV-2 infection, and 40 of them were synchronously regulated at both the mRNA and protein levels (Supplementary Fig. S6a, b). In the OB samples, 286 genes and 251 proteins were up/downregulated, and only four of them were consistently regulated at the mRNA and protein levels (Supplementary Fig. S6a, c). Gene enrichment analyses showed that SARS-CoV-2 infection induced a strong antiviral IFN response in the OE at 2 dpi, and the response decayed at 4 dpi, while no obvious changes were observed in the OB (Supplementary Fig. S7a). Further validation by qRT-PCR confirmed the results from RNA-seq analysis (Supplementary Fig. S7b, c). Notably, a strong inflammatory response in the OE was detected at both the mRNA and protein levels at 2 dpi, and this response faded at 4 dpi (Fig. 4a and Supplementary Fig. S8a). Moreover, genes related to “positive regulation of cell death” and “regulation of neuron projection development” were also upregulated upon SARS-CoV-2 infection (Fig. 4a and Supplementary Fig. S8b, c), which was consistent with the immunostaining results (Fig. 3d–f and Supplementary Fig. S5a, b). Further integrated omic analysis of the OE samples showed that a total of 30 genes were upregulated at both the mRNA and protein levels. Of these, antiviral genes/proteins, including Isg15, Stat1, Stat2, Oasl2, Ifit2, and Ifit3, were found to interact closely. Other genes/proteins involved in neurotransmitter transport, including Erc2, Lin7a, Slc1a3, and Slc25a18, were also observed (Fig. 4b). We did not find any obvious induction of antiviral response-related genes in OB samples by transcriptomic and proteomic analyses, but downregulation of inflammatory response-related genes was observed (Supplementary Fig. S9a, b).

Of particular note, KEGG pathway enrichment analysis of downregulated transcripts and proteins in the OE showed that genes belonging to “olfactory transduction” were significantly enriched (Fig. 4c). Among all 100 downregulated transcripts at 2 dpi, 36 encoded ORs (Fig. 4d and Supplementary Fig. S6b), while among the 278 downregulated transcripts at 4 dpi, 97 encoded ORs (Supplementary Figs. S6b and S8d). Further qRT-PCR assays showed that 13 representative OR genes were significantly downregulated in response to SARS-CoV-2 infection (Fig. 4e), which may also be attributed to the observed olfactory dysfunction. We further analyzed the expression of other host cell factors that were reported to facilitate SARS-CoV-2 infection in human cells. The results showed that Nrp1 was significantly downregulated at 2 dpi (Supplementary Fig. S10a), and slight downregulation of Bsg/CD147, Tmprss2, Furin, and Tfrc/transferrin receptor was also detected by RNA-seq analyses (Supplementary Fig. S10b–e).

## Discussion

In the present study, we used an established mouse model to demonstrate that SARS-CoV-2 infection can cause olfactory dysfunction and anosmia, and this experimental evidence supports the hypothesis that SARS-CoV-2 infection is the cause of olfactory dysfunction and anosmia in COVID-19 patients^5,6,22,23^. SARS-CoV-2-infected mice exhibited a damaged OE, immune cell infiltration, downregulated OR expression, and impaired olfactory function, largely mimicking the olfactory abnormalities of COVID-19 patients. Robust viral replication and direct antiviral responses were detected in the OE of the infected mice but not in the OB and other parts of the brain, indicating that SARS-CoV-2 mainly infected the OM in the hACE2 mouse model. A recent study also showed that the SARS-CoV-2 protein could be detected in the OE, but not in the OB, in a hamster model^17^. One possible explanation for the absence of SARS-CoV-2 in the CNS is that virus infection and replication in OE can effectively activate IFN-dependent antiviral responses (Supplementary Fig. S7a, b), which is an effective barrier that prevents viral invasion into the CNS. In addition, the apoptosis of infected OSNs may contribute to the prevention of viral spread into the CNS after the rapid infection and destruction of the OE^24^. Additionally, SARS-CoV-2 invaded brain tissues in K18-hACE2 mice, which expressed hACE2 under the cytokeratin 18 promoter^25^. These inconsistencies in SARS-CoV-2 infection outcomes, including CNS tropism, in different mouse models may be attributed to the different expression profiles of human ACE2 as well as the experimental systems.

Our results demonstrated that SARS-CoV-2 initially infects non-neuroepithelial cells, including sustentacular cells, Bowman’s gland cells and microvillar cells, which are involved in OSN support, host immune response, electrolyte balance maintenance, and mucus secretion^26^. Moreover, we observed various levels of damage in the OE after SARS-CoV-2 infection, including cilia desquamation, loss of surface microvilli, and substantial structural disorganization. In addition, our results showed a certain degree of cell apoptosis and inflammatory infiltration at both the cell and molecular levels following SARS-CoV-2 infection. All these data indicate that the damaged supporting non-neuroepithelial cells and inflammatory infiltration caused by SARS-CoV-2 infection contribute to the detrimental effects of the virus on olfactory function. Our results are supported by recent findings in mice and humans^15,25,27,28^, showing that the non-neuroepithelial cells of the OE express high levels of ACE2 and TMPRSS2 at both the mRNA and protein levels^15^. Interestingly, SARS-CoV-2-positive signals were also observed in HBCs and mOSNs of infected animals at 2 dpi and 4 dpi, respectively (Fig. 2a, c), although we did not detect any hACE2 expression in these cells (Supplementary Fig. S4d). The underlying mechanism remains elusive, and an hACE2-independent route of SARS-CoV-2 infection may be considered. According to recent studies, some other host cell receptors were found to facilitate SARS-CoV-2 infection, including NRP1, CD147, and transferrin receptor^29–32^. Additionally, SARS-CoV-2 contains a specific furin cleavage site in the spike protein^13,33^ and utilizes TMPRSS2 for further spike protein priming^14^. In our study, we found that the Nrp1, Bsg/CD147, Tmprss2, Furin, and Tfrc/transferrin receptor were downregulated to varying degrees in SARS-CoV-2-infected mice at 2 dpi, as shown by RNA-seq analyses, but whether these proteins play critical roles in olfactory dysfunction after SARS-CoV-2 infection in our animal model remains unknown and warrants extensive investigation.

We observed that many ORs were significantly downregulated at 2 and 4 dpi, suggesting the occurrence of decreased olfaction after SARS-CoV-2 infection. A recent study also showed that induction of antiviral type I interferon signaling in the mouse OE was associated with diminished odour discrimination and decreased RNA levels of ORs^34^. These findings may support what we observed in this study: SARS-CoV-2 infection causes a significant interferon response and dramatic OR decrease simultaneously in the OE. We also observed that the levels of three odorant-binding proteins (OBPs), which are compact globular water-soluble proteins with ligand-binding capabilities and are thought to aid in the capture and transport of odorants to the ORs, significantly decreased at the protein level with OE infection^35–37^. In addition, although no viral infection was observed in the OB, we detected some up/downregulated transcripts or proteins by transcriptomic and proteomic analyses. Notably, among all four proteins co-regulated at both the transcriptomic and proteomic levels, Rtp1 (receptor-transporting protein 1) was downregulated at both levels (Supplementary Table S1). This protein specifically promotes functional cell surface expression of ORs^38^, suggesting that the inhibition of Rtp1 in OB may lead to downregulation of ORs. Therefore, OE damage, which is closely related to olfactory dysfunction, is caused by SARS-CoV-2 infection of non-neuroepithelial cells and OSNs synergizes with the host antiviral immune responses.

According to our results, the olfactory dysfunction in SARS-CoV-2-infected animals was recoverable, as almost all animals recovered their normal sense of smell at 4 dpi. Additionally, studies focusing on COVID-19 patients with anosmia have shown that most of them recover from loss of smell within a few weeks or less, and only a small number of patients reported slow partial recovery over 1 to 3 months^39–42^, indicating a potential mechanism of OE regeneration from injuries. The hACE2 mouse model of SARS-CoV-2 infection reproduces the clinical course and viral replication observed in COVID-19 patients, as it mimics the acute SARS-CoV-2 infection as well as the transient olfactory dysfunction observed in human individuals. The difference in the time span of viral infection and clearance between humans and mice reflected the inherent differences between the species. The OE undergoes lifelong regeneration and replacement depending on two populations of basal stem cells, namely, HBCs and GBCs. HBCs are mitotically quiescent under normal conditions and become activated and differentiate into other kinds of cells once OE damage occurs^43^. Unlike HBCs, most GBCs are mitotically activated and are responsible for the regeneration of both neuronal and non-neuronal cells^44–46^. Indeed, we observed regeneration of the OE, as indicated by the significant proliferation and morphological change of HBCs, accompanied by the differentiation of stem cells into iOSNs, sustentacular cells and microvillar cells. In this way, the structural basis and function of the OE as well as olfactory function can be restored to normal in SARS-CoV-2-infected animals. Furthermore, it was indicated that the damage and apoptosis of OSNs are closely involved in their regeneration^47^, and the occurrence of an inflammatory response also facilitates stem cell differentiation and OE regeneration^48,49^. At the transcriptomic and proteomic levels, we observed upregulated “regulation of neuron projection development” genes/proteins at 2 and 4 dpi, implying the progression of a neuron projection over time from its formation to the mature structure. Interestingly, although there were many significantly downregulated ORs at 4 dpi, the mRNA levels of many ORs increased slightly compared with those at 2 dpi, indicating that OR expression tends to recover to normal levels.

In summary, our study established a mouse model of olfactory dysfunction induced by SARS-CoV-2. Considering the interspecies discrepancy in olfactory construction between rodents and humans, e.g., the relative size of the OB to the brain, the proportion of the brain involved in olfaction, and the expression of ORs^43^, further studies are recommended to reproduce SARS-CoV-2-induced olfactory dysfunction in other animal models, especially nonhuman primates. Additionally, validating the targets and biological effects of SARS-CoV-2 infection in human specimens should still be considered. An animal model of olfactory disorders is available to subsequently evaluate antiviral drugs as well as vaccines for the inhibition of SARS-CoV-2 and for the improvement of post viral olfactory disorders.

## Materials and Methods

### Cell and virus

Vero cells were maintained at 37 °C under 5% CO_2_ in Dulbecco’s modified Eagle’s medium (DMEM) supplemented with 10% heat-inactivated foetal bovine serum (FBS, Gibco), 10 mM HEPES, and 1% penicillin**/**streptomycin. The SARS-CoV-2 strain BetaCoV/Beijing/IMEBJ05/2020 (No. GWHACBB01000000) was originally isolated from a COVID-19 patient. For virus propagation, Vero cells were incubated with SARS-CoV-2, and the culture supernatants were collected at 3 dpi. The stock of SARS-CoV-2 was serially diluted and titrated on monolayers of Vero cells. Studies with infectious SARS-CoV-2 were conducted under biosafety level 3 (BSL3) facilities at the Beijing Institute of Microbiology and Epidemiology, AMMS.

### SARS-CoV-2 infection of hACE2 mice

The animal experiment procedure was reviewed and approved by the Laboratory Animal Center, AMMS (approval number: IACUC-DWZX-2020-001). For intranasal infection, 5.4 × 10^5^ PFU of SARS-CoV-2 was instilled into the nasal cavity of 6–8-week-old hACE2 mice anaesthetized with sodium pentobarbital at a dose of 50 mg/kg by the intraperitoneal route. Mice were monitored daily and euthanized at 2 or 4 dpi to isolate tissues.

### RNA extraction and qRT-PCR

Quantification of SARS-CoV-2 RNA, and OR mRNA transcript levels was performed by qRT-PCR. Total RNA was isolated using TRIzol reagent (Invitrogen, Carlsbad, CA, USA) according to the manufacturer’s instructions. SARS-CoV-2 RNA was measured with the following primer-probe set: CoV-F3 (5′-TCCTGGTGATTCTTCTTCAGGT-3′), CoV-R3 (5′-TCTGAGAGAGGGTC AAGTGC-3′) and CoV-P3 (5′-AGCTGCAGCACCAGCTGTCCA-3′). SARS-CoV-2 sgRNA was measured with the following primer-probe set: CoV-sgRNA-F (5′-CGATCTCTTGTAGATCTGTTCTC-3′), CoV-sgRNA-R (5′-ATATTGCAGCAGTACGCACACA-3′) and CoV-sgRNA-P (5′-ACACTAGCCATCCTTACTGCGCTTCG-3′)^3^. Glyceraldehyde-3-phosphate dehydrogenase (GAPDH) served as the endogenous control, and the following primer set was used: 5′-CCAACCGCGAGAAGATGA-3′ and 5′-CCAGAGGCGTACAGGGATAG-3′. The primer sequences for amplifying the OR genes are listed in Supplementary Table S2. Amplification was performed using a One Step PrimeScript RT-PCR Kit (Takara Bio, Otsu, Japan), and the following qRT-PCR conditions were applied: 42 °C for 5 min and 95 °C for 10 s followed by 40 cycles of 95 °C for 5 s and 60 °C for 20 s. The PCR was conducted in a LightCycler^®^ 480 Instrument (Roche Diagnostics Ltd, Rotkreuz, Switzerland). The absolute quantification of SARS-CoV-2 RNA levels was performed by comparison to a standard curve and is shown as SARS-CoV-2 RNA copies per mouse. The relative expression levels of OR mRNA were calculated according to the 2−ΔΔCt method. Each sample was assayed with three repeats.

### BFPT

The standard BFPT was used to evaluate the olfactory function of SARS-CoV-2-infected mice and DMEM-treated mice as previously described^50,51^. Mice were food-restricted to 0.2 g of chow per day for 2 days before the test and during the experimental period to ensure motivation. The food pellet was buried 1 cm below the surface of 3-cm-high bedding in a clear test cage (45 cm L × 24 cm W × 20 cm H). One mouse was placed in the centre of the cage, and the latency for the mouse to uncover the pellet was recorded. The latency was defined as 300 s for the mouse that could not find the pellet within 5 min.

### RNAscope ISH

RNAscope ISH for SARS-CoV-2 RNA was performed with the RNAscope assay (Advanced Cell Diagnostics, Newark, CA, USA) according to the manufacturer’s instructions. Briefly, the tissues were isolated immediately after euthanasia, fixed in 4% paraformaldehyde (PFA) for 24 h, and embedded in paraffin after being decalcified using 10% EDTA solution. Four-micrometre-thick formalin-fixed paraffin-embedded (FFPE) slides were warmed at 60 °C for 1 h before they were deparaffinized in xylene, rehydrated in a series of graded alcohols, and subjected to RNAscope target retrieval at 95 °C. Slides were visualized in situ using the 2.5 HD Reagent Kit (BROWN, Cat# 322310) and the sense probe from the RNAscope ISH probe-V-nCoV2019-S (Cat# 848561) at 40 °C in a HybEZ hybridization oven and then counterstained with hematoxylin.

### Multiplex immunofluorescent staining

The 4-μm-thick paraffin sections were deparaffinized in xylene and rehydrated in a series of graded alcohols. Antigen retrieval was performed in citrate buffer (pH = 6) by heating in a microwave (Sharp, R-331ZX) for 20 min at 95 °C followed by a 20 min cool-down period at room temperature. Multiplex fluorescence labeling was performed using TSA-dendron-fluorophores (NEON 9-color Allround Discovery Kit for FFPE, Histova Biotechnology, NEFP950). Briefly, endogenous peroxidase was quenched in 3% H_2_O_2_ for 20 min, followed by treatment with blocking reagent for 30 min at room temperature. Primary antibody was incubated for 2–4 h in a humidified chamber at 37 °C, followed by detection using the HRP-conjugated secondary antibody and TSA-dendron-fluorophores. Then, the primary and secondary antibodies were thoroughly eliminated by heating the slides in retrieval/elution buffer (Abcracker^®^, Histova Biotechnology, ABCFR5L) for 10 s at 95 °C using a microwave. In a serial fashion, each antigen was labeled with distinct fluorophores. The multiplex antibody panels applied in this study were as follows: hACE2 (Abcam, ab108209, 1:200); tdTomato (Rockland, 600-401-379, 1:500); SARS-CoV-2 nucleocapsid protein (Sinobiological, 40143-R004, 1:1000); GAP43 (Abcam, ab75810, 1:1000); OMP (Abcam, ab183947, 1:1500); CK5 (Abcam, ab52635, 1:800); CK8 (Abcam, ab53280, 1:800); Sox9 (Abcam, ab185230, 1:500); Sox2 (CST, 23064, 1:400); CD73 (CST, 13160, 1:500); Furin (Abcam, ab108209, 1:400); Tmprss2 (Abcam, ab92323, 1:500); CD3 (CST, 78588, 1:300); CD8 (CST, 98941, 1:300); Cleaved caspase-3 (CST, 9664, 1:1000); CD103 (Abcam, ab224202, 1:300); Ly-6G (CST, 87048, 1:400); CD68 (CST, 97778, 1:300); and Granzyme B (Abcam, ab255598, 1:300). After all the antibodies were detected sequentially, the slices were imaged using the confocal laser scanning microscopy platform Zeiss LSM880.

### Histopathological analysis

The structural integrity of the mouse OE was analyzed using H&E staining according to standard procedures. Briefly, after being rehydrated in a series of graded alcohols, 4-μm-thick slides of mouse OE were stained with hematoxylin for 30 s and washed in water. Slides were then stained in eosin for 15 s and washed again in water.

### RNA library construction and sequencing

hACE2 transgenic mice before or after SARS-CoV-2 infection (2 or 4 dpi) as previously described were used for RNA-seq. Total RNA from the OE and OB was extracted using TRIzol (Invitrogen, Carlsbad, CA, USA) and DNase I (NEB, USA), respectively. Sequencing libraries were generated using the NEBNext^®^ UltraTM RNA Library Prep Kit for Illumina^®^ (#E7530L, NEB, USA) following the manufacturer’s recommendations, and index codes were added to attribute sequences to each sample. Clustering of the index-coded samples was performed on a cBot cluster generation system using a HiSeq PE Cluster Kit v4-cBot-HS (Illumina, San Diego, California, USA) according to the manufacturer’s instructions. After cluster generation, the libraries were sequenced on the Illumina NovaSeq 6000 platform, and 150-bp paired-end reads were generated. After sequencing, a Perl script was used to filter the original data (raw data) to clean reads by removing contaminated reads for adapters and low-quality reads. Clean reads were aligned to the mouse genome (*Mus_musculus*.GRCm38.99) using Hisat2 v2.1.0. The number of reads mapped to each gene in each sample was counted by HTSeq v0.6.0, and the TPM (transcripts per kilobase of exon model per million mapped reads) was then calculated to estimate the expression levels of genes in each sample.

### Large-scale proteomic sample preparation and tandem mass tag (TMT) labeling

The OE and OB tissues were disrupted by using a grinding mill for six cycles of 5 s each with lysis buffer (9 M urea, 10 mM Tris-HCl, pH 8.0, 30 mM NaCl, 10 mM iodoacetamide (IAA), 5 mM Na_4_P_2_O_7_, 100 mM Na_2_HPO_4_, pH 8.0, 1 mM NaF, 1 mM Na_3_VO_4_, 1 mM sodium glycerophosphate, 1% phosphatase inhibitor cocktail 2 (Sigma, St. Louis, USA), 1% phosphatase inhibitor cocktail 3 (Sigma, St. Louis, USA), and 1 tablet of EDTA-free protease inhibitor cocktail (Roche, Basel, Switzerland) for every 10 mL of lysis buffer) and 2-mm steel balls, respectively. The supernatants were obtained after centrifugation at 8000 rpm for 10 min at 4 °C. The protein lysates were inactivated at 56 °C for 30 min and then stored at –80 °C before further processing. The protein concentration was measured by performing a short Coomassie blue-stained 10% SDS-PAGE run as previously described^52^. The same amount of protein (130 μg) from each sample was reduced with 5 mM dithiothreitol (DTT), alkylated with 20 mM IAA, and precleaned by 10% SDS-PAGE (10%, 0.7 cm). The protein was then subjected to in-gel digestion with a final concentration of 12.5 ng/μL Ac-trypsin combined with endoproteinase lys-C provided by Enzyme & Spectrum (Beijing, China) at a ratio of 2:1 at 37 °C for 12–14 h^53,54^. The extracted peptides from the OE and OB groups were labeled with TMT10 reagents according to the manufacturer’s instructions (Thermo Scientific, San Jose, CA, USA). Ten labeled channels were then quenched with 5% hydroxylamine and combined according to the normalization value by ratio checking. The mixed samples were vacuum dried.

### Peptide fractionation and LC-MS/MS analysis

The dried TMT-labeled mixture was resuspended in 100 μL of buffer A (2% acetonitrile (ACN), pH 10) and separated by a high-pH reversed-phase HPLC system (Rigol, L-3120, Beijing, China). The combined samples were injected into a Durashell C_18_ column (150 Å, 5 μm, 4.6 × 250 mm^2^) and eluted with a linear gradient in 60 min. Briefly, the solvent gradient of buffer B (2% ddH_2_O and 98% ACN) was as follows: 0% for 5 min, 0–3% for 3 min, 3%–22% for 37 min, 22%–32% for 10 min, 32%–90% for 1 min, 90% for 2 min, and 100% for 2 min. The LC flow rate was set at 0.7 mL/min and monitored at 214 nm. The column oven was set at 45 °C. A total of 60 fractions were collected and then combined into 15 fractions before vacuum drying according to the peak abundance. The combined samples were dissolved in loading buffer (1% ACN and 1% formic acid (FA)) and analyzed using an EASY-nLC 1200 ultra-performance liquid chromatography system (Thermo Fisher Scientific, San Jose, CA, USA) equipped with a self-packed capillary column (75 μm i.d. × 15 cm, 3 μm C_18_ reversed-phase fused silica), with a 78-min nonlinear gradient at a flow rate of 600 nL/min. The gradient was as follows: an increase from 6%–15% solvent B (0.1% FA in 80% ACN) in 15 min, 15%–30% in 40 min, 30%–40% in 15 min, and 40%–100% in 1 min, finally holding at 100% for 7 min. The eluted peptides were analyzed on an Orbitrap Fusion Lumos (Thermo Fisher Scientific, San Jose, CA, USA). MS_1_ data were collected in the Orbitrap using a 120 k resolution over an *m/z* range of 300–1500, with the maximum injection time (MIT) set to 50 ms. The automatic gain control (AGC) was set to 4 × 10^5^, determined charge states between 2 and 7 were subjected to fragmentation via higher energy collision-induced dissociation (HCD) with 37% collision energy, and a 12 s dynamic exclusion window was used with isotopes excluded. For the MS/MS scans, the fractions were detected in the Orbitrap at a resolution of 50 k. For each scan, the isolation width was 1.6 *m/z*, the AGC was 5 × 10^4^, and the MIT was 86 ms.

### Database search

The raw files from the OE and OB groups were searched with MaxQuant (v1.5.5.0) against the mouse reviewed proteome downloaded from UniProt, containing 17,478 entries and a canonical SARS-CoV-2 proteome with 30 potential viral proteins from the SARS-CoV-2 genome (NC_045512.2), and a common contaminant database (http://www.maxquant.org/contaminants.zip), respectively. Fully tryptic peptides with as many as 2 missed were allowed. TMT 10 plex (N-Term/K) and cysteine carbamidomethyl were set as fixed modifications, whereas oxidation of methionine was set as a variable modification. The tolerance of the precursor and fragment ions was set to 20 ppm.

### Bioinformatic analyses

DESeq2 v1.6.3 was used for differential gene expression analysis. Genes with *P*adj ≤ 0.05 and |Log_2_FC | > 1 were identified as differentially expressed genes (DEGs). The total proteomic quantification datasets were median-normalized, and *P* value was calculated by Perseus (1.6.6.0). Proteins ratios between control and infection ≥ 1.5-fold and *P* value ≤ 0.05 were considered as regulated differentially expressed proteins (DEPs). The DEGs and DEPs identified were used as queries to search for enriched biological processes (Gene Ontology BP) using Metascape^55^. KEGG pathway enrichment and protein interaction networks were analyzed using STRING^56^. Heatmaps of gene expression levels were constructed using the pheatmap package in R (https://cran.rstudio.com/web/packages/pheatmap/index.html). Dot plots and volcano plots were constructed using the ggplot2 (https://ggplot2.tidyverse.org/) package in R.

### Statistical analysis

Data were analyzed using GraphPad Prism 8 (GraphPad Software, San Diego, California, USA). The values shown in the graphs are presented as means ± SD of at least three independent experiments. Statistical differences between groups were analyzed using two-tailed unpaired *t*-tests or a one-way ANOVA statistical test with Dunnett’s multiple comparison test; *P* < *0.05* was considered statistically significant.

## Supplementary information

Supplementary Information

## Data Availability

All the RNA-seq data from this study have been deposited to the NCBI Gene Expression Omnibus (GEO) datasets under accession number GSE173186. The raw MS/MS data of the proteomes have been deposited to the ProteomeXchange Consortium (http://proteomecentral.proteomexchange.org) via the iProX partner repository^57^. The accession number for the MS/MS data reported in this paper is the iProX (https://www.iprox.org/) dataset identifier IPX0002998000.
