# Supplementary material for: SARS-CoV-2 infection in the mouse olfactory system
Source: Cell Discov. 2021 Jul 6;7:49. doi: 10.1038/s41421-021-00290-1 (PMC8260584; doi:10.1038/s41421-021-00290-1)
Supplement: Supplementary file 1 — Supplementary Information [file 41421_2021_290_MOESM1_ESM.pdf]

**Supplementary Information**

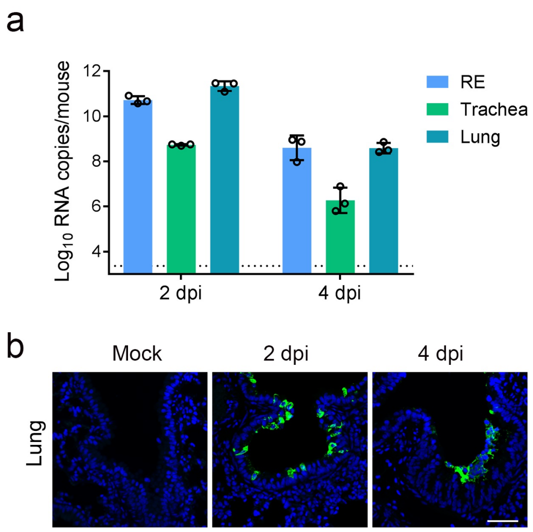


**Supplementary Fig. S1 SARS-CoV-2 infection in the respiratory tract of hACE2 mice. a**, Viral RNA detection in the nasal RE, trachea and lung of SARS-CoV-2-infected mice. Viral RNA copies were determined by qRT-PCR and are shown as the mean ± SD from three independent replicates. **b**, Representative immunostaining results for lung tissue from SARS-CoV-2-infected or mock-treated mice for SARS-CoV-2 N protein (green) and DAPI (blue). Scale bar, 50 μm.

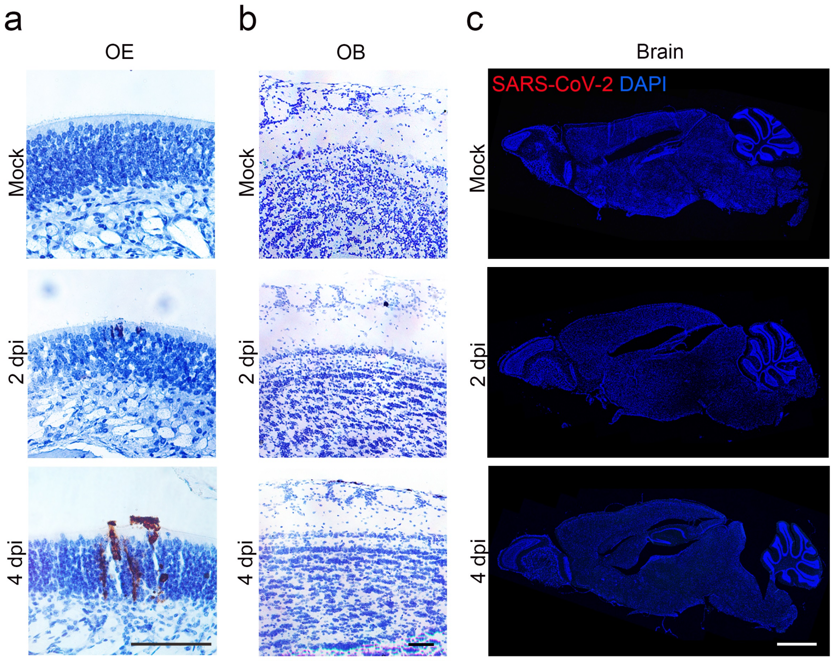


**Supplementary Fig. S2 SARS-CoV-2 infection in the olfactory system of hACE2 mice. a**, **b**, Representative results for RNAscope *in situ* hybridization (ISH) for SARS-CoV-2 RNA detection in the OE (**a**) or OB (**b**) tissue from SARS-CoV-2-infected or mock-treated mice. Scale bar, 100 μm. **c**, Representative immunostaining results for brain tissues from SARS-CoV-2-infected and mock-treated mice for SARS-CoV-2 N protein (green) and DAPI (blue). Scale bar, 2 mm.

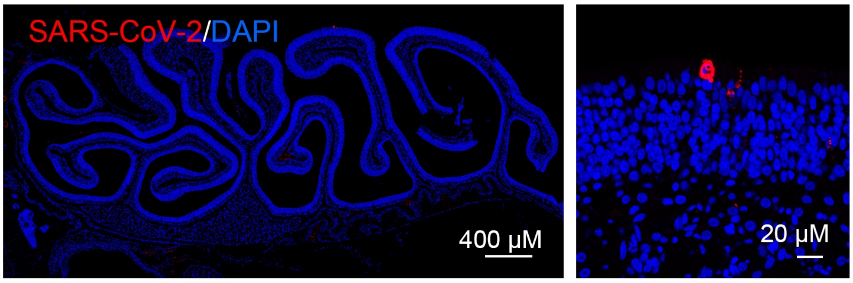


**Supplementary Fig. S3 Representative immunostaining results for the OE from SARS-CoV-2-infected mice at 8 dpi for SARS-CoV-2 N protein (red) and DAPI (blue).**

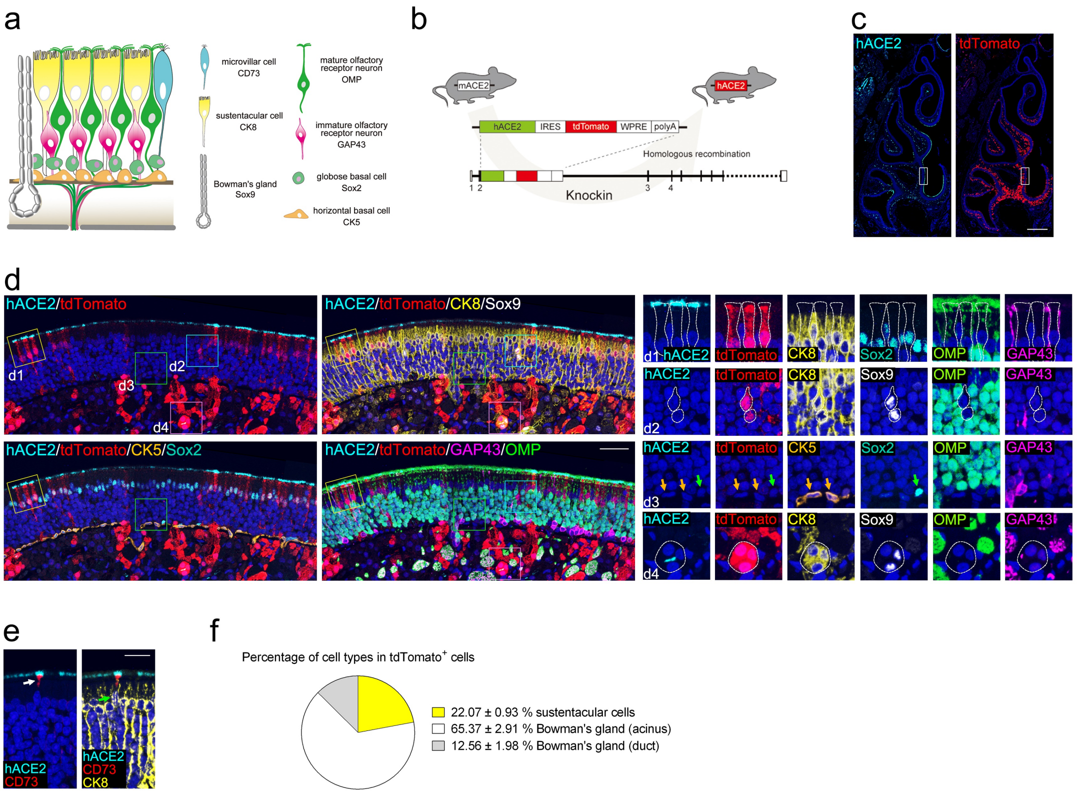


**Supplementary Fig. S4 hACE2 is mainly expressed by non-neuroepithelial cells in the OE of hACE2 mice**. **a**, Schematic diagram showing the cell compartments of the mouse OE and their representative markers. **b**, Schematic diagram showing the strategy of generating hACE2 mice by insertion of a hACE2-IRES-tdTomato cassette into exon 2 of the endogenous mouse ACE2 gene. **c**, Representative immunofluorescent staining of hACE2 (cyan) and tdTomato (red) in coronal sections of the hACE2 mouse olfactory system. The framed area is shown in **d** at larger magnifications with multiple cell markers, including CK8 (yellow), Sox9 (white), CK5 (gold), Sox2 (emerald), GAP43 (magenta) and OMP (green). The framed areas in **d** are labelled d1 (the surface region of the OE), d2 (the duct of Bowman’s gland), d3 (the basal region of the OE) and d4 (the acinus of Bowman’s gland) and are shown adjacently at larger magnifications with each marker separately displayed. The tdTomato+ cells are outlined by dashed lines. None of the HBCs or GBCs expressed hACE2 or tdTomato (gold and green arrows, respectively). **e**, Representative multiplex immunofluorescent staining results showing that CD73/CK8-positive microvillar cells also expressed hACE2. **f**, Statistical analysis

of the percentage of each cell compartment within the tdTomato-positive cells. The data are presented as the mean ± SD (n = 3). Scale bar, 500 μm in **c**; 50 μm in **d**, **e**.

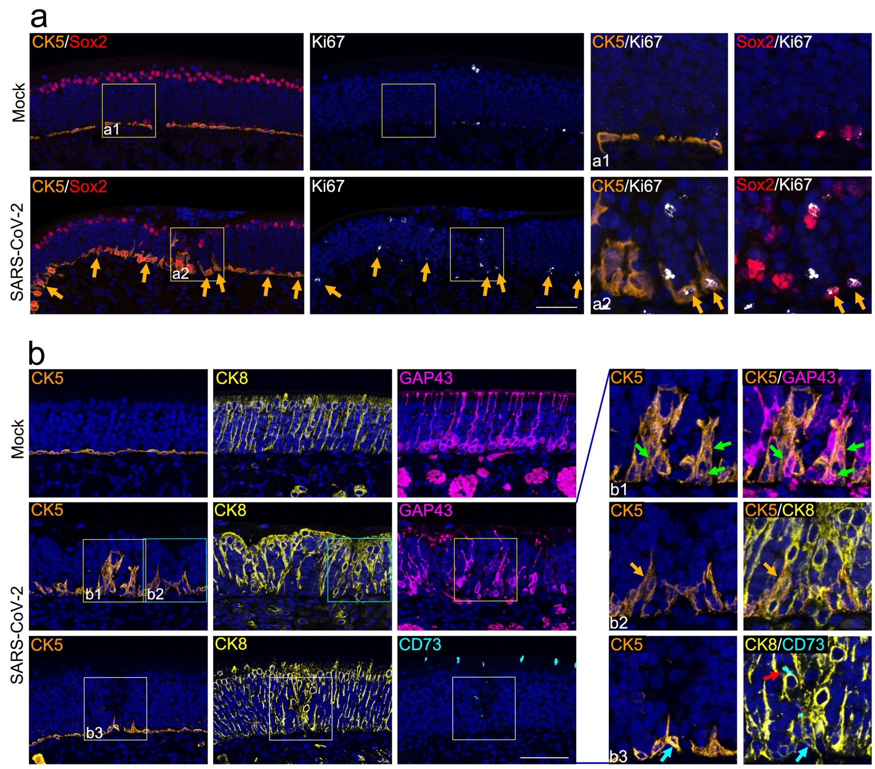


**Supplementary Fig. S5 SARS-CoV-2 infection triggers regeneration of the OE. a**, Representative results for immunofluorescent staining of CK5 (gold), Sox2 (red) and Ki67 (white) showing the increase in actively cycling olfactory stem cells, labelled CK5/Sox2/Ki67 triple-positive, after infection (gold arrows). The framed areas labelled a1 and a2 are shown adjacently at larger magnifications. **b**, Representative immunofluorescent staining of CK5 (gold), CK8 (yellow), CD73 (cyan) and GAP43 (magenta) shows the transition states during the differentiation of HBCs. The framed areas labelled b1–b3 are shown adjacently at larger magnifications. Green arrows in b1 denote CK5/GAP43 double-positive cells. Gold arrows in b2 denote CK5/CK8 double-positive cells. Cyan arrows and red arrows in b3 denote CK5/CK8 and CK8/CD73 double-positive cells, respectively. Scale bar, 50 μm.

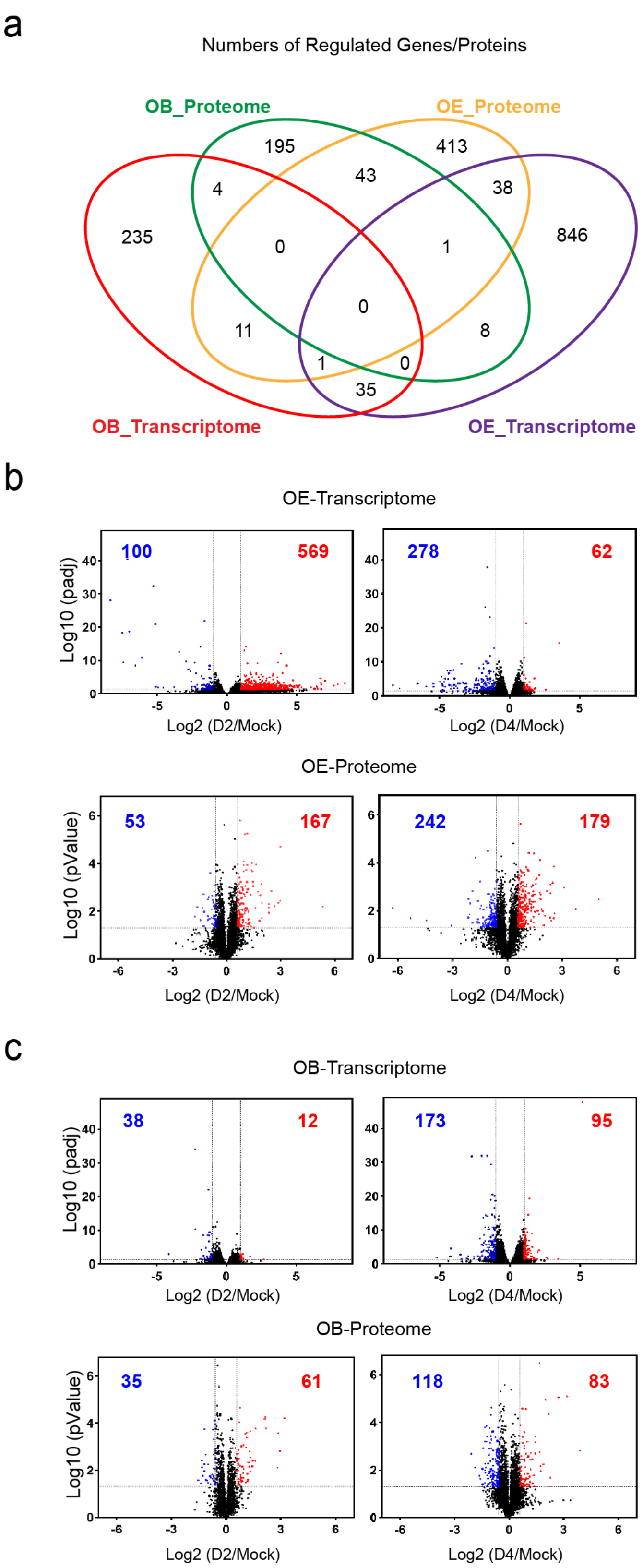


**Supplementary Fig. S6 Comparison of regulated genes or proteins corresponding to SARS-CoV-2 infection in OE and OB. a**, Comparison of regulated genes or

proteins in SARS-CoV-2-infected OE and OB. The Venn diagram depicts genes or proteins that were shared and/or unique in each comparison. **b**, Volcano plots indicating differentially regulated genes and proteins of the OE during the course of SARS-CoV-2 infection. **c**, Volcano plots indicating differentially regulated genes and proteins of the OB during the course of SARS-CoV-2 infection. Upregulated genes (padj $< 0.05$) with a $\log_2$ (fold change) of more than 1 are indicated in red, and downregulated genes (padj $< 0.05$) with a $\log_2$ (fold change) of less than -1 are indicated in blue. Upregulated proteins ($P$ value $< 0.05$) with a $\log_2$ (fold change) of more than 0.58 are indicated in red, and downregulated proteins ($P$ value $< 0.05$) with a $\log_2$ (fold change) of less than -0.58 are indicated in blue.

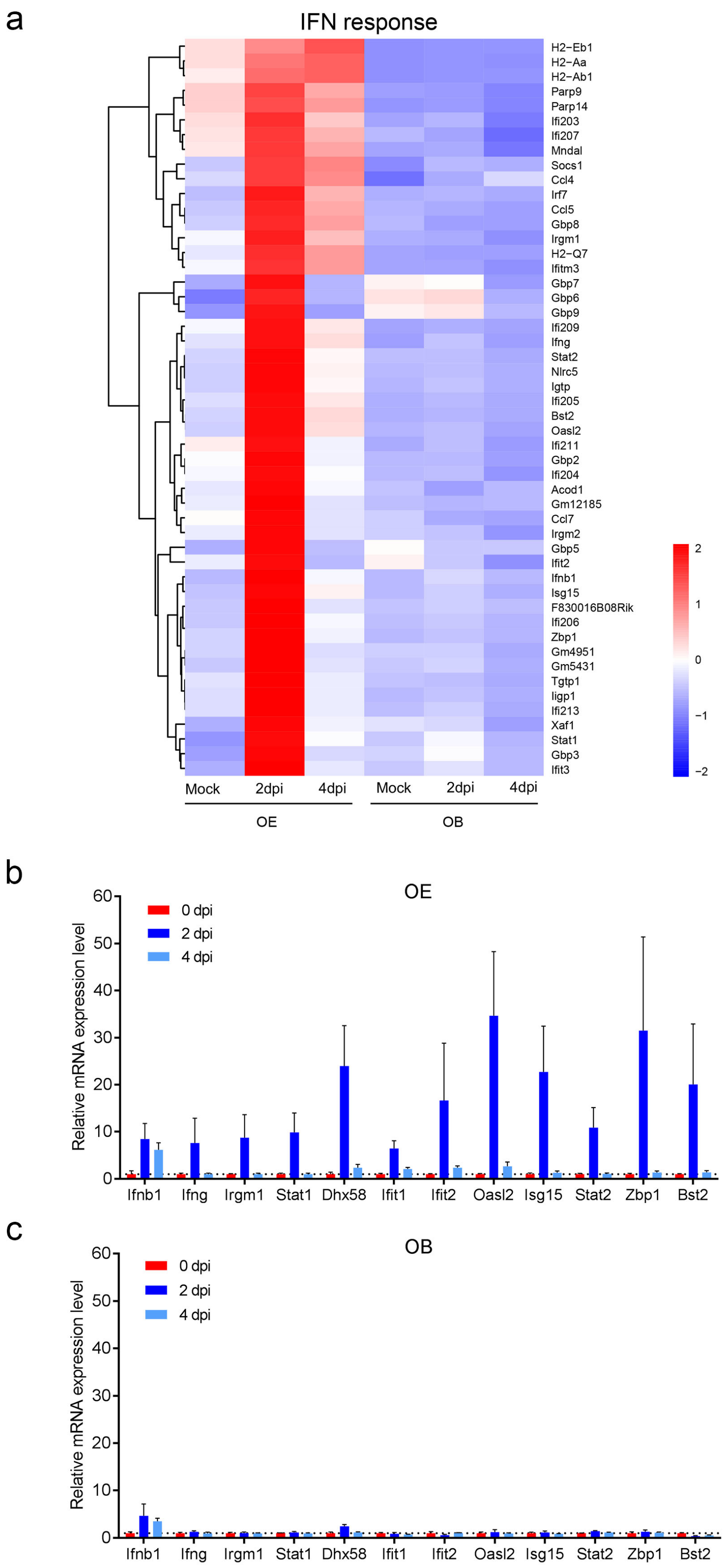
a
IFN response
H2−Eb1
H2−Aa
H2−Ab1
Parp9
Parp14
Ifi203
Ifi207
Mndal
Socs1
Ccl4
Irf7
Ccl5
Gbp8
Irgm1
H2−Q7
Ifitm3
Gbp7
Gbp6
Gbp9
Ifi209
Ifng
Stat2
Nlrc5
Igtp
Ifi205
Bst2
Oasl2
Ifi211
Gbp2
Ifi204
Acod1
Gm12185
Ccl7
Irgm2
Gbp5
Ifit2
Ifnb1
Isg15
F830016B08Rik
Ifi206
Zbp1
Gm4951
Gm5431
Tgtp1
Iigp1
Ifi213
Xaf1
Stat1
Gbp3
Ifit3
2
1
0
−1
−2
Mock
2dpi
4dpi
Mock
2dpi
4dpi
OE
OB
b
OE
0 dpi
2 dpi
4 dpi
Relative mRNA expression level
60
50
40
30
20
10
0
Ifnb1
Ifng
Irgm1
Stat1
Dhx58
Ifit1
Ifit2
Oasl2
Isg15
Stat2
Zbp1
Bst2
c
OB
0 dpi
2 dpi
4 dpi
Relative mRNA expression level
60
50
40
30
20
10
0
Ifnb1
Ifng
Irgm1
Stat1
Dhx58
Ifit1
Ifit2
Oasl2
Isg15
Stat2
Zbp1
Bst2

**Supplementary Fig. S7 Antiviral IFN responses to SARS-CoV-2 infection in the OE and OB. a,** Heatmaps depicting the type-I and type-II IFN responses in the OE and OB. The coloured bar represents the Z-score of the TPM. **b, c,** RNA expression of Ifnb1/IFN-β, Ifng/IFN-γ and ISGs, as determined by qRT-PCR.

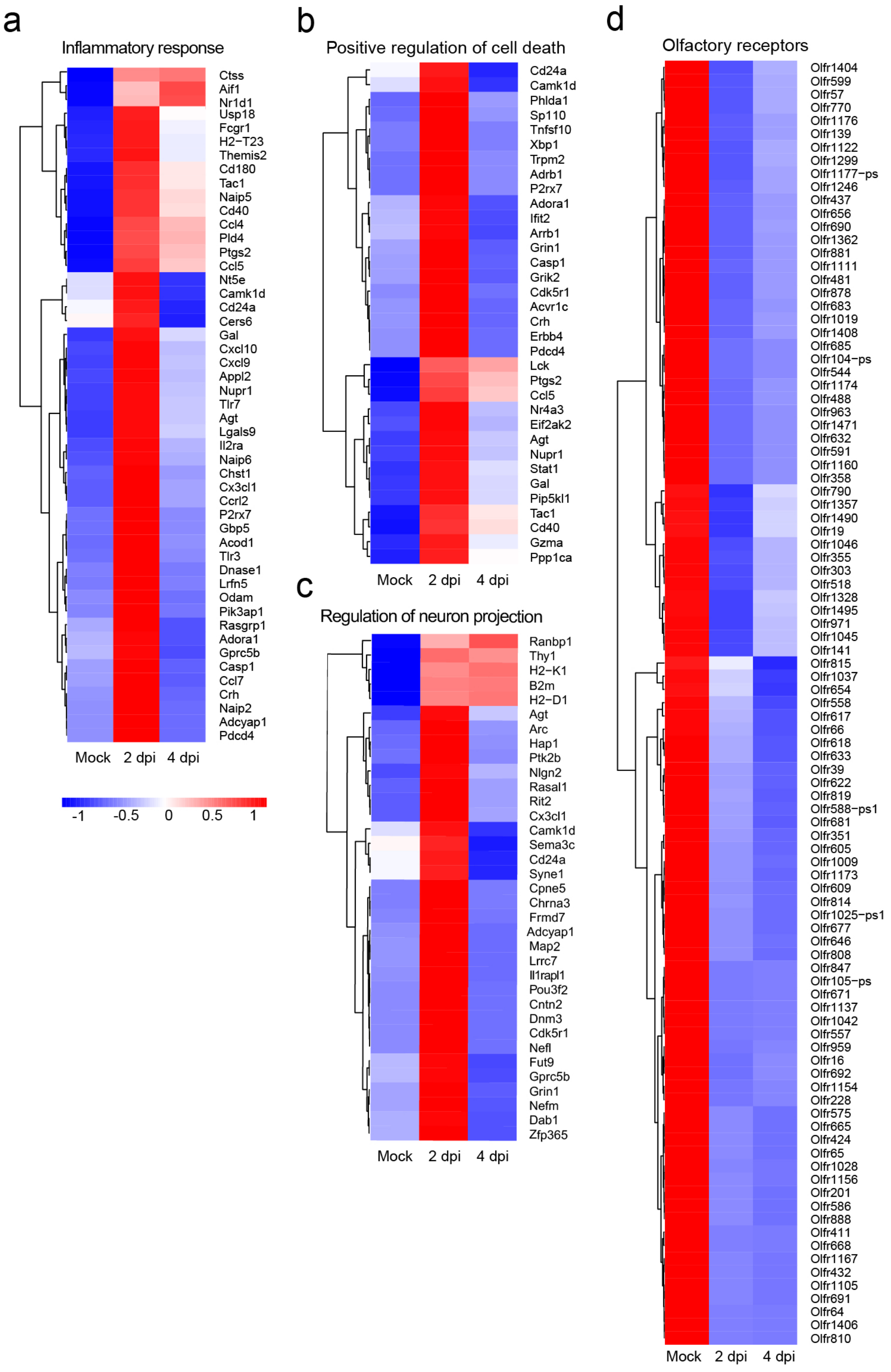


**Supplementary Fig. S8 Heatmaps depicting the expression levels of the regulated genes enriched in different GO-annotated groups in the OE. a**, Inflammatory response (GO:0006954). **b**, Positive regulation of cell death (GO:0010942). **c**, Regulation of neuron differentiation (GO:0045664). The graphs depict the DEGs of the infected OE compared with the mock-treated OE at 2 and/or 4 dpi. The genes included

have a | $\log_2$(fold change) | of more than 1 and a padj value of less than 0.05. **d**, Heatmap indicating the expression patterns of 97 olfactory receptor genes that were significantly downregulated at 4 dpi. The coloured bar represents the Z-score of the TPM.

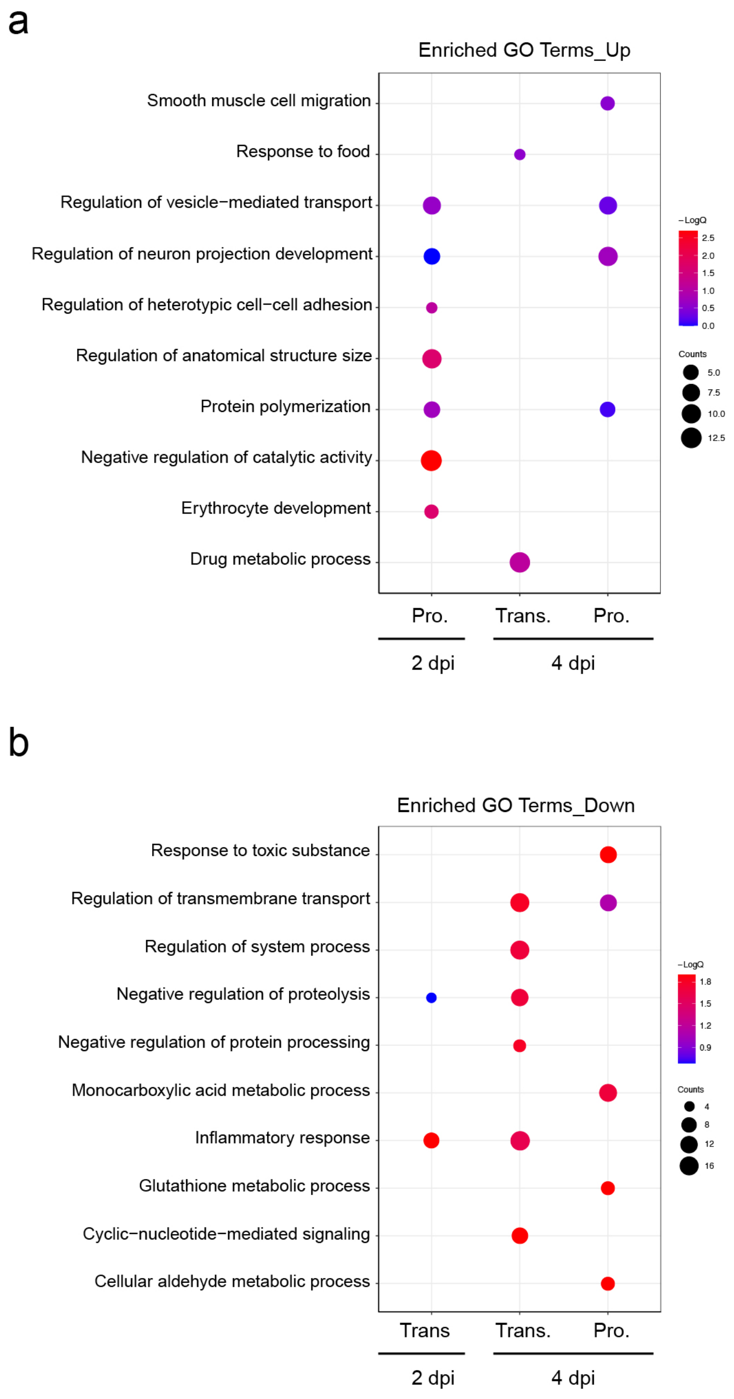


**Supplementary Fig. S9 Host response to SARS-CoV-2 in the OB at the mRNA and protein levels. a**, Dot plot visualization of enriched GO terms of upregulated genes/proteins at 2/4 dpi in the OB. No GO terms were enriched using upregulated genes at 2 dpi. **b**, Dot plot visualization of enriched GO terms of downregulated genes/proteins at 2/4 dpi in the OB. No GO terms were enriched using upregulated

proteins at 2 dpi. Gene enrichment analyses were performed using Metascape against the GO dataset for biological processes.

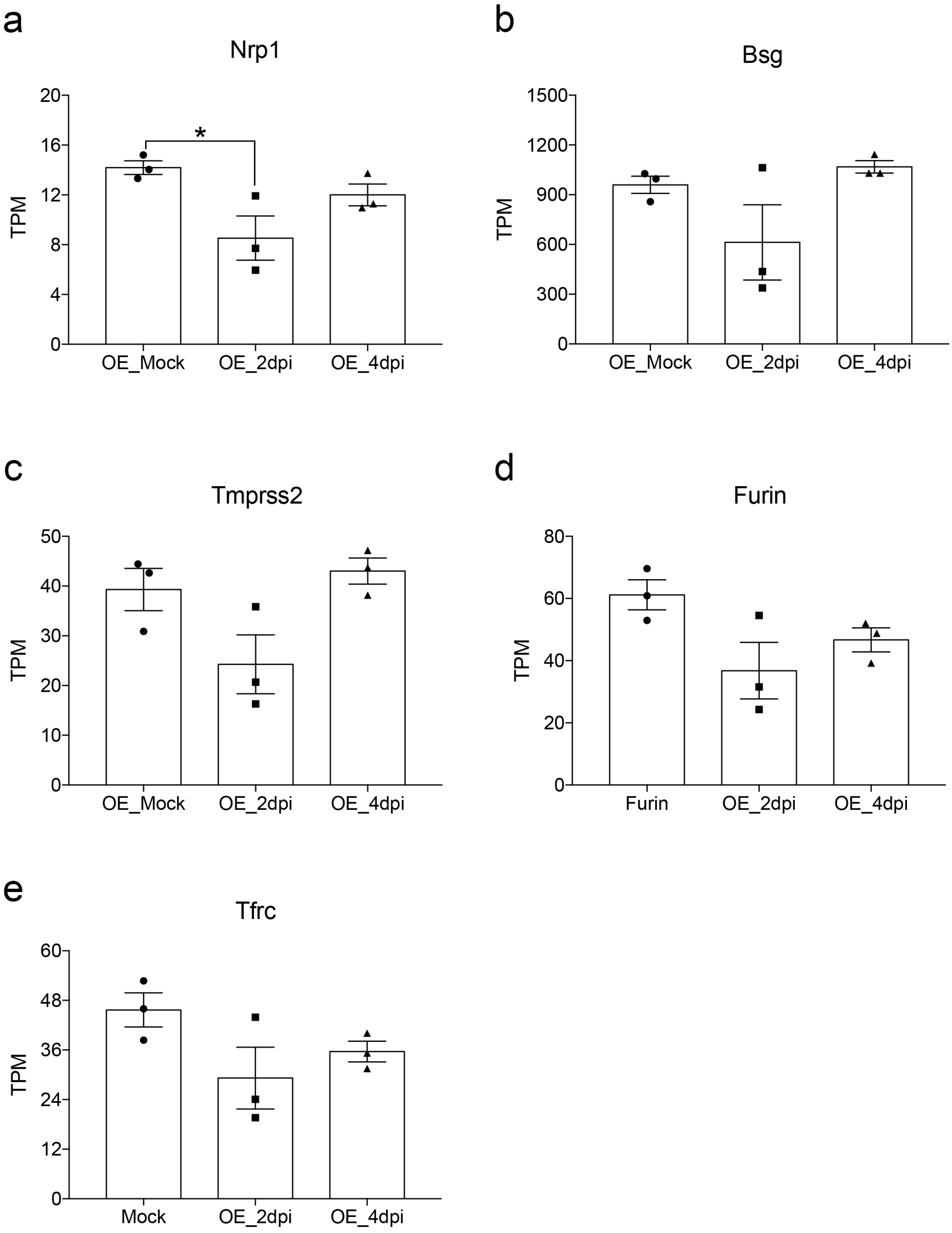


**Supplementary Fig. S10 RNA expression levels of Nrp1 (a), Bsg/CD147 (b), Tmprss2 (c), Furin (d) and the Tfrc/transferrin receptor (e) by RNA-seq analyses.** Columns with * indicate significant downregulation at $P < 0.05$ relative to their mock groups (one-way ANOVA followed by post hoc analysis with Dunnett's multiple comparisons test, n = 3).

**Supplementary Table S1. Detailed information of 4 proteins co-regulated at both transcriptomic and proteomic level along the course of SARS-CoV-2 infection in OB.**

| Gene symbol | Transcriptome | Proteome | Description |
|---|---|---|---|
| | Mock 2dpi 4dpi | Mock 2dpi 4dpi | |
| Rtp1 | 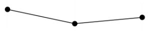 | 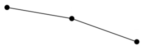 | Receptor-transporting protein 1; Specifically promotes functional cell surface expression of olfactory receptors, but not of other GPCRs |
| Clca3a1 | 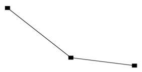 | 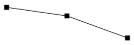 | Calcium-activated chloride channel regulator 3A-1; Plays a role in modulating chloride current across the plasma membrane in a calcium-dependent manner |
| Cbr2 | 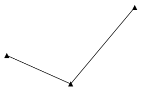 | 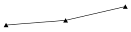 | Carbonyl reductase [NADPH] 2; May function in the pulmonary metabolism of endogenous carbonyl compounds, such as aliphatic aldehydes and ketones derived from lipid peroxidation, 3-ketosteroids and fatty aldehydes, as well as in xenobiotic metabolism |
| Mt3 | 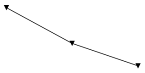 | 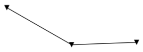 | Metallothionein-3; Binds heavy metals. Contains three zinc and three copper atoms per polypeptide chain and only a negligible amount of cadmium. Inhibits survival and neurite formation of cortical neurons in vitro (By similarity) |

**Supplementary Table S2. Primers used for qRT-PCR. F: forward strand; R: reverse strand.**

| Name | Sequence |
|---|---|
| mOlfr959 forward primer | TCCAGTCGGCTTCATACCC |
| mOlfr959 reverse primer | ACACTCGGTACAACCAAGGAA |
| mOlfr57 forward primer | CTGTGGATTGATGATTCTGGCA |
| mOlfr57 reverse primer | TGGGGGATTTCCAAGTTTGTG |
| mOlfr1046 forward primer | ATGGAGAAATCCAACCACAGTG |
| mOlfr1046 reverse primer | GTTTCGAGTCTACATGAGTCAGG |
| mOlfr971 forward primer | GTTCACAGTGGGAGTTTACACTT |
| mOlfr971 reverse primer | GCAGGTGTCAAGATATTCAGTGC |
| mOlfr145 forward primer | TGTGCTTACTCCTTTTGCTAGG |
| mOlfr145 reverse primer | CTGGTGCAAGAGCGTTCCA |
| mOlfr1045 forward primer | TCCTGGTTTTCATTGTAGCAGAG |
| mOlfr1045 reverse primer | AAGCCATAGAAGTATGTGAGGGA |
| mOlfr1137 forward primer | GGTGTGAAAGTGGTTTTGTTTGT |
| mOlfr1137 reverse primer | CAGCATCCTGGGTCCAACT |
| mOlfr19 forward primer | GCCGCCTCTGTGGATTTTTAG |
| mOlfr19 reverse primer | ACAGGCAAGTAGTACCAGTTGA |
| mOlfr1195 forward primer | GTGAGTTTCCTTATGCTCATGGT |
| mOlfr1195 reverse primer | CTTGTCCATAGGGAAGGTGGT |
| mOlfr1389 forward primer | ACTTTCAATGCCAGTTTAGGGAA |
| mOlfr1389 reverse primer | TGCCAAGGAGAGTTAGCAGAT |
| mOlfr134 forward primer | TGTGTCCTCCTGGCTCTTATGTCC |
| mOlfr134 reverse primer | AGTGGCCTCTGAGACAGCATAGG |
| mOlfr1065 forward primer | CGCAAGGCTTTCTCCACCTGTG |
| mOlfr1065 reverse primer | TGACTGGATTTGGGCTGCATGTAC |
| mOlfr738 forward primer | ATCATGGGGTCTTATGCTCTAGT |
| mOlfr738 reverse primer | GCTGCATGTTCAGATGTTGGG |
| Dhx58 forward primer | GGAAGTGATCTTACCTGCTCTGG |

| | |
|---|---|
| Dhx58 reverse primer | TTGCCTCTGTCTACCGTCTCT |
| Ifih1 forward primer | AGATCAACACCTGTGGTAACACC |
| Ifih1 reverse primer | CTCTAGGGCCTCCACGAACA |
| Zbp1 forward primer | AAGAGTCCCCTGCGATTATTTG |
| Zbp1 reverse primer | TCTGGATGGCGTTTGAATTGG |
| Bst2 forward primer | TGTTCGGGGTTACCTTAGTCA |
| Bst2 reverse primer | GCAGGAGTTTGCCTGTGTCT |
| Irgm1 forward primer | TGCTCCACTACTCCCCAACAT |
| Irgm1 reverse primer | GCTCCTACTGACCTCAGGTAAC |
| Stat1 forward primer | TCACAGTGGTTCGAGCTTCAG |
| Stat1 reverse primer | GCAAACGAGACATCATAGGCA |
| Stat2 forward primer | TCCTGCCAATGGACGTTCG |
| Stat2 reverse primer | GTCCCACTGGTTCAGTTGGT |
| Isg15 forward primer | GGTGTCCGTGACTAACTCCAT |
| Isg15 reverse primer | TGGAAAGGGTAAGACCGTCCT |
| Ifit2 forward primer | AGTACAACGAGTAAGGAGTCACT |
| Ifit2 reverse primer | AGGCCAGTATGTTGCACATGG |
| Oasl2 forward primer | TTGTGCGGAGGATCAGGTACT |
| Oasl2 reverse primer | TGATGGTGTCGCAGTCTTTGA |
| Ifnb1 forward primer | CAGCTCCAAGAAAGGACGAAC |
| Ifnb1 reverse primer | GGCAGTGTAACTCTTCTGCAT |
| Ifng forward primer | ATGAACGCTACACACTGCATC |
| Ifng reverse primer | CCATCCTTTTGCCAGTTCCTC |
